# Impact of Maternal Body Mass Index on Maternal and Neonatal Outcomes Among Sudanese Women

**DOI:** 10.7759/cureus.18365

**Published:** 2021-09-28

**Authors:** Rayan A Eltayeb, Amani A Khalifa

**Affiliations:** 1 Obstetrics and Gynaecology, Khartoum University, Khartoum, SDN; 2 Obstetrics and Gynaecology, Woman Wellness Research Center, Hamad Medical Corporation, Doha, QAT

**Keywords:** bmi, pregnancy, neonate, nutrition, complications

## Abstract

Background: Unhealthy weight has an adverse impact on a person’s general health, this is more serious for pregnant a woman as it can affect her baby as well. Nutrition is an important element of antenatal care. Nutrition must be optimum to avoid problems of being underweight, and that of overweight. This study aims to assess the impact of maternal body mass index on maternal and neonatal outcomes among Sudanese women.

Methods: A cross-sectional study was carried out at Saad Abu El Ella Hospital and Soba University Hospital in December 2017. The maternal weight and height were measured for 159 women to calculate BMI. The impact of maternal BMI on maternal and neonatal outcomes was tested using the chi-square test and logistic regression. P-value = or <0.05 was considered as statistically significant.

Results: Fourty-two (26.4%) of studied women were of normal weight, two (1.3%) were underweight, 61 (38.4%) were overweight and 54 (33.9%) were obese. Increasing parity was associated with higher BMI, this was more significant among the obese group with an odds ratio of 3.1 (95% CI = 1.099-8.866, p=0.033). There was a relative increase in the risk of delivery by caesarean section among the obese group with an odds ratio of 1.7 (95% CI =1.079-2.737, p=0.023). No significant association between maternal BMI and preterm or post-term delivery was found. The incidence of macrosomia was more among overweight and obese mothers. There was no association found between maternal BMI and low birth weight, admission of the baby to neonatal intensive care unit (NICU), or low Apgar score at 5 minutes.

Conclusion: This study shows a high incidence of being overweight among Sudanese pregnant ladies. Increased risk of caesarean section as maternal BMI increases. Overweight and obese mothers are more likely to have macrosomic babies.

## Introduction

Pregnancy is an important event during a woman’s life. It has a great impact on her physical and mental health. All efforts that are carried out during pregnancy aim to ensure full-term pregnancy without complications, safe delivery, and a healthy baby. Special care should be given to nutrition as a part of antenatal care. Adequate nutrition is required to avoid problems that result from weight extremes. Weight gain during pregnancy was assessed based on body mass index (BMI) before pregnancy. Mothers with low BMI must gain more weight than those with high BMI. Appropriate gaining of weight during pregnancy is important to maternal and baby’s long-term health. The National vital statistic system birthdate 2015 showed that about 32% of women in the USA gained recommended amount of weight during pregnancy, while 21% gained too little and 48% gained too much [1]. 

Despite the fact that obesity is a leading cause of morbidities such as diabetes, cardiovascular diseases, and musculoskeletal problems, it is considered a sign of female beauty in Sudanese culture. A cross-sectional study in four universities in Khartoum, Sudan showed that women try to gain weight by taking tablets, injections (insulin), herbal, and high-calorie diets (source: submission for the degree of Master of Philosophy by Ibtisam Ismail Ahmed, Mar 2012; available at https://pure.southwales.ac.uk/files/2270708/I._I._Ahmed_2012_2060265.pdf).

It is noticed that normal-weight mothers are less likely to develop complications than underweight, overweight, and obese mothers, hence the efforts to encourage ladies to keep their weight within normal range before, during, and after pregnancy. Statistics showed that out of a total of 128,290 women who reported having given birth in the UK during March-April 2009, 6413 were identified as obese at any time during pregnancy, the UK prevalence rate was 4.99% [2].

In Sudan, a study carried out during February-April 2008 found that among 1690 pregnant ladies, 94 (5.5%) were underweight, 603 (35.6%) were overweight, and 328 (19.4%) were obese [3].

Special care should be provided to underweight, overweight, and obese mothers since they are more susceptible to pregnancy and delivery complications. According to WHO underweight mothers are at higher risk to develop intrauterine growth restriction. A study conducted at Swansea University showed that ''costs to NHS per pregnancy were 23% more for overweight women and 37% more for obese'' [4].

Information about lifestyle changes, especially that’s of eating habits should be provided to ladies during antenatal care, since ladies are more prepared to change their habits to keep their babies healthy. A healthy lifestyle education is vital and effective before conception, a survey of women’s attitudes, concerning healthy lifestyle changes prior to pregnancy, showed that 70% of the women said that they changed their lifestyle before they became pregnant, and the health professionals were found to be the most effective information source in convincing ladies to make these health changes [5]. Providing such information must be based on figures; hence, the aim of this study is to assess the impact of maternal body mass index on maternal and neonatal outcomes among Sudanese women.

## Materials and methods

This descriptive cross-sectional study was conducted in Saad Abu El Ella Hospital and Soba University Hospital, over a period from 1st of December to 31st of December 2017. Institutional Review Board at the Department of Community Medicine, University of Khartoum issued approval KU\FM\CM\Y-5. The ethical clearance of this study was obtained from the Department of Community Medicine at the University of Khartoum, Soba University Hospital's director, and Saad Abu El Ella Hospital's director. Verbal consent was taken from each lady participant in the study before filling the checklist.

The study population was all Sudanese pregnant ladies who were admitted for delivery to Saad Abu El Ella Hospital, or Soba University Hospital over a period from 1st of December to 31st of December 2017. The study sample of 159 women was taken by a simple random sampling method. Women were excluded if they had multiple pregnancies, known co-morbidity (diabetes, hypertension, liver, renal or cardiac diseases), or if they refused to participate in the study.

 A designed checklist was used in data collection. It was developed after an extensive literature review. A pilot checklist was tested on a convenience sample of 10 ladies in Ibrahim Malik Hospital. The checklist involved four sections. The first section was the demographic data which included seven questions about: maternal age, parity, occupation, monthly income, education, delivery spacing, and contraceptive use. The second section was the BMI measurement which included height, weight, calculated BMI, and BMI categories. The third section was the maternal outcome variables that included two questions about the mode of delivery and pregnancy complications, either medical such as diabetes, hypertension, anaemia, or obstetric such as preterm delivery and post-term delivery. The fourth section was the neonatal variables which included four questions about baby’s sex, birth weight, Apgar score at 5 minutes, and admission to the neonatal intensive care unit (NICU). Information was obtained by interviewing the participants and reviewing the records. Weight and height were measured by a well-titrated electronic platform scale, and tape. Weight (kg) and height (meter) were measured in light clothing without shoes while standing straight, then body mass index (BMI) was calculated as weight (kg) divided by height square (m^2^). BMI was used to classify women according to WHO’s classification as: underweight (BMI<18.5 kg/m^2^), normal (BMI 18.50 - 24.99 kg/m^2^), overweight (BMI 25 - 29.99 kg/m^2^), and obese (BMI >30 kg/m^2^). Data were analyzed in four groups according to the BMI categories: normal, underweight, overweight, and obese. Data were analysed with SPSS statistics version 23.0 (IBM Corp., Armonk, NY). Dichotomous data were presented as counts or frequencies and continuous variables summarized as means (±SD). Variables were presented as frequency and percentage. Chi-square test and logistic regression were used to compare frequencies and percentages between BMI categories. Statistical significance was set as P-value = or <0.05.

## Results

One hundred and fifty-nine women, who had a singleton delivery, were included in this study. The women were between 18 and 43 years old, their mean (±SD) age was 29.32(±5.9) years. Among them 30.8% were primiparous and 69.2% were multiparous, the mean (±SD) parity of them was 2.9(±1.9). 69.8% of the studied women were from urban areas while 30.2% of them were from rural areas. Participants coming from small families were 41.5% and 58.5% were from extended families. Regarding the occupation of participants, 74.8% were housewives, 20.8% were working outside their houses (different professions), and 4.4% were students. Contraceptive methods among ladies were as follows: 18.9% used oral contraceptives, 2.5% used injections, 3.1% used implants, 1.3% used a contraceptive intrauterine device (IUD), and 10.1% used other methods. The remaining 64.2% did not use any contraceptive methods. The distribution of BMI was as follows: 42 (26.4%) were of normal weight (BMI 18.50 - 24.99 kg/m^2^), two (1.3%) were underweight (BMI <18.5 kg/m^2^), 61 (38.4%) were overweight (BMI 25 -29.99 kg/m^2^), and 54 (33.9%) were obese (BMI > 30 kg/m^2^). Increasing parity was associated with higher BMI, this was more significant among the obese group with an odds ratio (OR) of 3.1 (95% CI = 1.099-8.866, p=0.033).

Out of 159 studied women: 115 (72.3%) delivered by cesarean section and the remainder 44 (27.7%) delivered by normal vaginal delivery. An association between maternal BMI and mode of delivery was found (p=0.011). As there was a relative increase in the risk of delivery by caesarean section among the obese group with OR 1.7 (95% CI =1.079-2.737, p=0.023). Observed pregnancy complications were: diabetes (3.1%), hypertension (8.8%), anaemia (2.5%), infection (3.77%), bleeding (0.63%), deep venous thrombosis (0.63%), polyhydramnios (3.14%), oligohydramnios (0.63%), preterm delivery (9.4%), and post-term delivery (3.1%) (Table 1).

**Table 1:** Relation between maternal BMI and pregnancy outcomes among Sudanese ladies in Saad Abu El Ella Hospital and Soba University Hospital in December 2017.

Variable	Normal weight (n=42)	Underweight (n=2)	Overweight (n=61)	Obese (n=54)	P value
Preterm delivery	3(7.1%)	1(50%)	6(9.8%)	5(9.3%)	.332
Post-term delivery	2(4.8%)	0	3(4.9%)	0	.332
Diabetes	2(4.8%)	0	3(4.9%)	0	.275
Hypertension	2(4.8%)	0	2(3.3%)	10(18.5%)	.275
Anaemia	2(4.8%)	0	2(3.3%)	0	.275
Others	2(4.8%)	0	3(4.9%)	3(5.6%)	.275
Normal vaginal delivery	17(40.5%)	2(100%)	15(24.9%)	10(18.5%)	.011
Caesarean section	25(59.5%)	0	46(75.4%)	44(81.5%)	.011

Out of 159 babies, five died perinatally. Among the live newborn, the male to female ratio was 85:69. Twenty-one (13.6%) of them were of low birth weight, six (3.9%) were macrosomic and the remaining 127 (82.5%) were of normal weight. Thirteen (8.4%) babies had a low Apgar score at 5 minutes. Fourty-one (26.6%) babies were admitted to NICU, the causes of admission were: jaundice (24.4%), infection (22%), respiratory problems (19.5%), aspiration (17.1%), prematurity (14.6%), hypoglycemia (7.3%), hydrocephalous (4.9%), spina bifida (2.4%), and trauma (2.4%). Incidences of macrosomia were more among overweight and obese mothers. There was no association between maternal BMI and low birth weight, admission of the baby to neonatal intensive care unit (NICU), or low Apgar score at 5 minutes. Maternal BMI and neonatal outcomes are illustrated in Table 2.

**Table 2:** Relation between maternal BMI and neonatal outcomes among Sudanese ladies in Saad Abu El Ella Hospital and Soba University Hospital in December 2017. NICU - Neonatal Intensive Care Unit

Neonatal variable	Normal weight (n=42)	Underweight (n=2)	Overweight (n=61)	Obese (n=54)	Total (n=159)	P valve
Low birth weight	6(14.3%)	1(50%)	6(9.8%)	8(14.8%)	21(13.2%)	.315
Macrosomia	0	0	2(3.3%)	4(7.4%)	6(3.8%)	.315
Low Apgar score	4 (9.5%)	0	6(9.8%)	3(5.6%)	13(8.2%)	.823
Admission to NICU	12(28.6%)	2(100%)	15(24.6%)	12(22.2%)	41(25.8%)	.117
Fetal mortality	0	0	2(3.3%)	3(5.6%)	5(3.1%)	.482

## Discussion

Like most of the similar studies [6-10], this study estimated nutritional status by measuring BMI which is considered the best method, some studies used body weight and mid-arm circumference data [11,12].^ ^BMI, also known as Quetelet index, is defined as a person's weight in kilograms divided by the square of the person's height in meters (kg/m^2^). It was developed by Adolphe Quetelet during the 19th century as an inexpensive and easy method for assessing nutritional status [13]. BMI is better than other body fatness measures like skin fold thickness, dual-energy X-ray absorptiometry (DXA), underwater weighing, bioelectrical impedance, and isotope dilution. BMI is an easily available, standardized, inexpensive evaluation that does not need to be conducted by highly trained personnel [14]. BMI before conception or at early first trimester appears to be the most helpful measurement for maternal nutritional status, but this was not possible in our study as it was a cross-sectional study in one month and the majority of the women in Sudan do not record their weight before pregnancy or even have it measured at the antenatal clinic. Instead of calculating BMI at early pregnancy, we calculated it immediately after delivery which is also considered an effective parameter.

Unlike two Nigerian studies [15,16], this study found that increasing parity is associated with higher BMI, this was more significant among the obese group (OR 3.1 ''1.099-8.866''), (p=.033), similar to other studies [17-19].

Advanced maternal age is associated with increased risk for obesity [17-19],^ ^but some studies showed no association between maternal BMI and age [15,16]; however, our study didn't prove this association. Although lyoke et al.'s study [16], mentioned that occupation and education level are different between healthy and obese women, in Chigbu and Aja's study [15], as well as in this study, occupation, and education did not have significant relation with BMI. In contrast to other studies [15,16],^ ^residency was similar between BMI groups.

Davies and colleagues in their study mentioned that low-income is a risk for being underweight [20],^ ^this was not approved here, maybe due to the small size of the underweight group. Like other studies [21-23],^ ^an association between maternal BMI and mode of delivery was present. This study found that obese mothers are more likely to have caesarean section, which has more complications than normal delivery. Furthermore, Edomwonyi and Osaigbovo’s study shows more intraoperative complications in obese parturients than in non-obese [24]. Some studies mentioned that preterm delivery is associated with obesity [8,10,21], while others found that preterm delivery is more among underweight mothers [7,25]. In our study as well as in another study carried out in Khartoum teaching hospital [6],^ ^no association between maternal BMI and preterm delivery existed.

Mothers with higher BMI have an increased risk of post-term delivery [26],^ ^this is not approved in our study, may be due to ethnic differences as that study was from a European country and also different method, as they reported the first trimester BMI. Admission of babies to NICU and low Apgar score at 5 minutes appeared to have no relation with maternal BMI in our study and in Sahu et al.'s study [9], as both studies were carried out in tertiary hospitals they differ from another community-based study which found this relation [27]. Unlike Sahu et al.'s study [9], we found that maternal BMI does not affect birth weight.

A limitation of this study is being a cross-sectional study it did not allow following up babies during the neonatal period, and as a result, some conditions that may be related to their prenatal environment which, of course, are affected by maternal weight, might be missed.

## Conclusions

This study shows a high incidence of overweight among Sudanese pregnant ladies. The study also finds that the more the lady gains weight the higher the risk to deliver by caesarean section. Furthermore, overweight and obese mothers are more likely to have macrosomic babies. Therefore, it is recommended to carry out more efforts to increase the awareness of ladies about the importance of maintaining a healthy weight. This should be done by physicians, magazines, television programs, websites, and other ways of mass media.

Also, more researches should be done in the area of pregnancy-associated nutrition in Sudan such as awareness of ladies about normal weight and good nutrition during pregnancy, and the role of physicians at antenatal care in increasing awareness about nutrition and related issues, detecting nutrition-associated disorders, and helping to maintain normal weight gain throughout pregnancy.

## Appendices

                                                          Community medicine department

                                                                    Faculty of medicine

                                                                  University of Khartoum

Impact of maternal body mass index on maternal and neonatal outcomes among Sudanese women in Saad Abu EL Ella and Soba university hospital, December 2017

Check list number:

-Section one (demographic information):

1-Maternal age:                

2-Parity:

3-Last menstrual period:                  

4-Monthly income:

5-Occupation:

6-Education:

7-Residency:1-Urban     

                      2-Rural

8-Birth spacing:

9- Contraception methods:

-Section two:

1-Weight:

2-Height:

3-Body mass index (BMI):

4-BMI classification:

- Section three (maternal variables):

a-Pregnancy complications:

1-Diabetes                    

2-Hypertension                  

3-Anaemia         

4-Pre-term delivery            

5-Posterm pregnancy   

6-Others           

7-None

b-Mode of Delivery: 1-Normal vaginal delivery 

                                  2- Cesarean section

-Section four (neonatal variables):

1-Gender:

2-Birth weight:

3-Apgar score at 5 minutes:

4- Admission to the neonatal intensive care unit: 1-Yes            

                                                                                  2-No

5-If yes, cause of admission:
